# Dissemination of IMP-4-encoding pIMP-HZ1-related plasmids among *Klebsiella pneumoniae* and *Pseudomonas aeruginosa* in a Chinese teaching hospital

**DOI:** 10.1038/srep33419

**Published:** 2016-09-19

**Authors:** Wei Feng, Dongsheng Zhou, Qian Wang, Wenbo Luo, Defu Zhang, Qiang Sun, Yigang Tong, Weijun Chen, Fengjun Sun, Peiyuan Xia

**Affiliations:** 1 Department of Pharmacy, Southwest Hospital, the Third Military Medical University, Chongqing 400038, China; 2 State Key Laboratory of Pathogen and Biosecurity, Beijing Institute of Microbiology and Epidemiology, Beijing 100071, China; 3 College of Food Science and Project Engineering, Bohai University, Jinzhou 121013, China; 4 Beijing Institute of Genomics, Chinese Academy of Sciences, Beijing, 100029, China

## Abstract

A total of 26 *bla*_IMP-4_-carrying strains of *Pseudomonas aeruginosa* and *Klebsiella pneumoniae* were isolated from 2009 to 2013 in a Chinese teaching hospital, and these strains can be assigned into multiple sequence types or allelic profiles as determined by multilocus sequence typing. Of these strains, *P. aeruginosa* P378 and *K. pneumoniae* 1220 harbor the IMP-4-encoding plasmids pP378-IMP and p1220-IMP, respectively, whose complete nucleotide sequences are determined to be genetically closely related to the IncN1-type plasmid pIMP-HZ1. pP378-IMP/p1220-IMP-like plasmids are hinted to be present in all the other *bla*_IMP-4_-carrying strains, indicating the dissemination of pIMP-HZ1-related plasmids among *K. pneumoniae* or *P. aeruginosa* of different genotypes in this hospital. pP378-IMP carries two distinct accessory resistance regions, a *bla*_IMP-4_-carrying class 1 integron In*823b*, and a truncated Tn*3*-family unit transposon ΔTn*6292*-3′ harboring the quinolone resistance gene *qnrS1*. Massive fragmentation and rearrangement of these accessory genetic contents occur among p1220-IMP and IMP-HZ1 relative to pP378-IMP*. bla*_IMP-4_ is also present in the In*823b* remnants from p1220-IMP and IMP-HZ1, while *qnrS1* is located in a Tn*6292*-derive fragment from pIMP-HZ1 but not found in p1220-IMP. pP378-IMP represents the first fully sequenced IncN-type plasmid from *P. aeruginosa*.

Plasmids belonging to the IncN incompatibility group commonly have broad host range and high transmission efficiency, and they are important to the dissemination of clinically important resistance determinants among enterobacterial species. Location of the major carbapenem resistance genes such as *bla*_IMP_^1^, *bla*_KPC_^2^, *bla*_NDM_^3^ and *bla*_VIM_^4^ have been found on differen IncN-type plasmids. The IncN plasmids can be further divided into three subgroups, namely IncN1to IncN3, with their reference plasmids R46 (accession number AY046276), p271A^5^ and pN-Cit^6^, respectively. These three different plasmid subgroups have similar plasmid scaffolds but limited nucleotide sequence similarity over their backbones^5^,^6^.

The IMP-type enzymes are among the clinically most important metallo-β-lactamase and can hydrolyze almost all β-lactams including carbapenems. The first IMP-type enzyme IMP-1 was described in 1991 in Japan from *Serratia marcescens*^7^ and, since then, at least 52 IMP-variant enzymes (http://www.ncbi.nlm.nih.gov/projects/pathogens/beta-lactamase-data-resources/) have been reported worldwide among Enterobacteriaceae, *Acinetobacter*, and *Pseudomonas* species. The *bla*_IMP_ genes are commonly located on a plasmid-borne class 1 integrons, which are critical for the acquisition, maintenance, and dissemination of resistance in gram-negative organisms^8^.

Up to now, a total of three *bla*_IMP_-carrying IncN1 plasmids, namely pKPI-6^1^, and pIMP-HZ1^9^ and its isoform pIMP-1495 (GenBank accession number KM977631), all of which are recovered from *Klebsiella pneumoniae*, have been fully sequenced. pIMP-HZ1/pIMP-1495 and pKPI-6 harbor the *bla*_IMP-4_ and *bla*_IMP-6_ genes captured by two distinct class 1 integrons In*823* and In*722*, respectively.

This study provides the evidence for dissemination of *bla*_IMP-4_-carrying pIMP-HZ1-related plasmids among *K. pneumoniae* or *P. aeruginosa* strains of different genotypes from 2009 to 2013 in a Chinese public hospital. The whole genome sequences of pP378-IMP and p1220-IMP from two of these strains are determined to be genetically closely related to the IncN1-type plasmid pIMP-HZ1. pP378-IMP contains a *bla*_IMP-4_-carrying integron In*823b* and a truncated Tn*3*-family unit transposon ΔTn*6292*-3′ harboring *qnrS1*; by contrast, massive fragmentation of In*823b* and Tn*6292* and further complex rearrangement of the relevant fragments occur in p1220-IMP (containing *bla*_IMP-4_ and *qnrS1*) and pIMP-HZ1 (containing only *bla*_IMP-4_). Denoting dramatic genetic variations in the accessory resistance regions among these three plasmids.

## Results and Discussion

### bla_*IMP-4*_ *-carrying* K. pneumoniae and P. aeruginosa *isolates*

**Figure 1:**
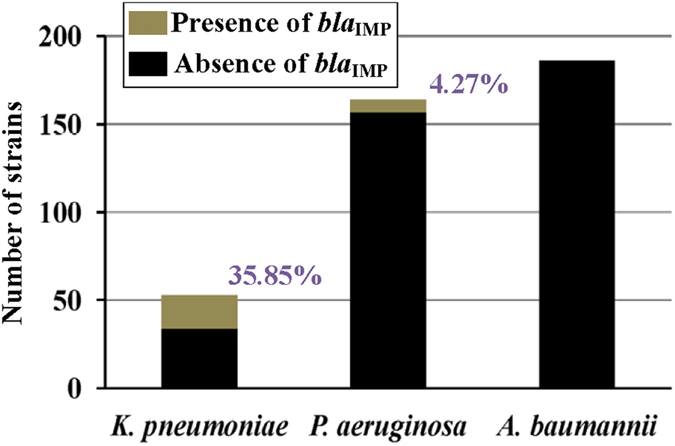
Prevalence of blaIMP among imipenem-nonsusceptible bacterial isolates. The *bla*_IMP_ genes are screened by PCR^21^, followed by amplicon sequencing. All the detected *bla*_IMP_ markers are the *bla*_IMP-4_ gene.

From 2009 to 2013, a total of 403 imipenem-nonsusceptible strains of *K. pneumoniae* (53 strains), *P. aeruginosa* (164 strains) and *A. baumannii* (186 strains) were isolated from the patients (with infections at various sites of their bodies) from our hospital (Fig. 1). Presence of *bla*_IMP_ was detected by PCR in 19 (35.85%) strains of *K. pneumoniae* and in 7 (4.27%) strains of *P. aeruginosa* (Fig. 1), and all these detected *bla*_IMP_ genes were *bla*_IMP-4_ as further determined by sequencing. None of the *A. baumannii* strains tested by PCR harbored the *bla*_IMP_ marker (Fig. 1).

These *bla*_IMP-4_-carrying *K. pneumoniae* and *P. aeruginosa* isolates (Table S1), scattered from 2009 to 2013, came from five distinct specimens (sputum, lung lavage fluid, wound secretion, urine, and blood) from eight different departments (Department of Pediatrics, Department of Pediatric ICU, Department of Respiratory Medicine, Department of Neurology, Department of Neurosurgery, Department of Cerebral Surgery, Department of Nephrology, and Department of emergency).

As determined by multilocus sequence typing (MSLT), the *bla*_IMP-4_-positve *K. pneumoniae* strains could be assigned into six different sequence types (STs), namely ST37 (allelic profile: 2-9-2-1-13-16-1), ST107 (2-1-2-17-27-39-1), ST133 (12-1-1-2-5-36), ST323 (2-1-1-1-9-93), ST686 (4-1-1-3-3-54), and ST1114 (4-3-2-1-10-17) (Table S1). The *bla*_IMP-4_-positve *P. aeruginosa* strains could be assigned into at least four allelic profiles, namely 15-?-1-4-11-4-10, 6-?-4-3-11-4-7, 2-?-5-1-3-6-11, 111-?-64-30-26-59-7, but unfortunately they could not be assigned into any of known or novel STs because the *aroE* sequences (corresponding to ‘?’ in the allelic profiles) for all the strains tested could not be obtained with repeated attempts (Table S1). The above results indicated the non-clonal dissemination of *bla*_IMP-4_-carrying *K. pneumoniae* and *P. aeruginosa* in the hospital.

### pP378-IMP and p1220-IMP from *P. aeruginosa* and *K. pneumoniae*

Two *bla*_IMP-4_-positive strains, *P. aeruginosa* P378 isolated from the urine specimen of a 36-year-old male with urinary tract infection and consciousness disturbance, and *K. pneumoniae* 1220 from the blood specimen of a three-month-old baby boy with neonatal septicemia and hyperbilirubinemi, were arbitrarily selected for transferring the *bla*_IMP-4_ marker into *E. coli* EC600 through conjugation, generating the *bla*_IMP-4_-positive *E. coli* transconjugants P378-IMP-EC600 and 1220-IMP-EC600, respectively. All these four strains had the class B carbapenemase activity and were resistant to piperacillin, piperacillin/tazobactam, cefazolin, cefuroxime, ceftazidime, cefepime, imipenem, and meropenem; moreover, P378 and P378-IMP-EC600, but not 1220 and 1220-IMP-EC600, were resistant to ciprofloxacin and levofloxacin (Table 1). Taken together, either *P. aeruginosa* P378 or *K. pneumoniae* 1220 harbors a conjugative *bla*_IMP-4_-carrying plasmid, designated pP378-IMP and p1220-IMP, respectively, which account for the carbapenem resistance phenotype.

**Figure 2:**
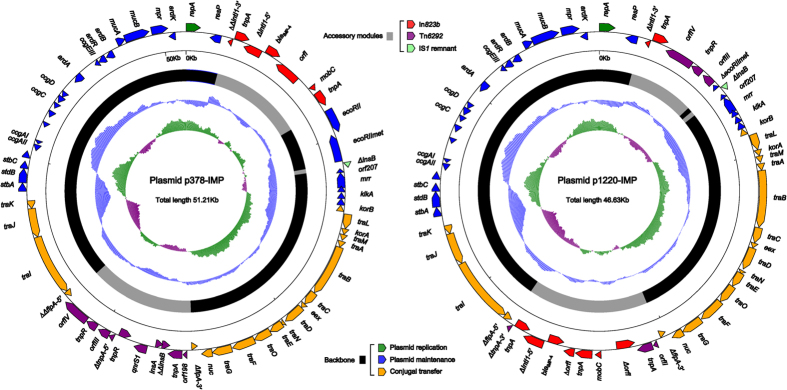
Schematic maps of sequenced plasmids. Genes are denoted by arrows and colored based on gene function classification. The innermost circle presents GC-Skew [(G–C)/(G + C)] with a window size of 500 bp and a step size of 20 bp. The blue circle presents GC content. Shown also are backbone and accessory module regions.

Whole-genome sequencing of pP378-IMP and p1220-IMP (with mean coverage >80), recovered from the P378-IMP-EC600 and 1220-IMP-EC600 strains, respectively, showed that these two plasmids have circularly closed DNA sequences, 51,207 bp and 46,629 bp in length, respectively (Fig. 2). pP378-IMP and p1220-IMP have mean GC contents of 50.5% and 50.7% and contain 64 and 60 predicted open reading frames in total, respectively (Fig. 2).

### Backbones of pP378-IMP and p1220-IMP

The entire sequences of pP378-IMP and p1220-IMP are mostly similar to that of pIMP-HZ1 (>99% query coverage and maximum >99% nucleotide identity). pP378-IMP, p1220-IMP and pIMP-HZ1 possess the conserved IncN1-type backbone regions, which contain a *repA* gene and its iterons (RepA-binding sites; regulation of replication) for plasmid replication, the *tra* genes and *kikA*-*korB* for conjugal transfer, the CUP (conserved upstream repeat) -controlled regulon, the *stbABC*-*orfD* operon, and *resP*) for plasmid maintenance (Fig. 2). These backbone regions are highly similar to the IncN1 prototype plasmid R46 from *Salmonella enterica* serovar Typhimurium.

**Figure 3:**
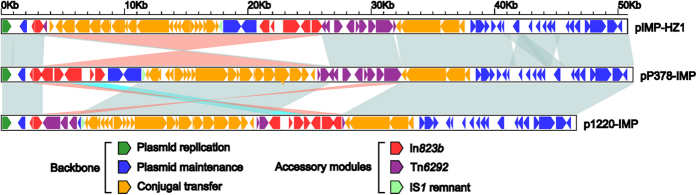
Linear comparison of sequenced plasmids. Genes are denoted by arrows and colored based on gene function classification. Shading regions denote regions of homology (>95% nucleotide identity).

There are four major genetic differences among the backbones of pP378-IMP, p1220-IMP and IMP-HZ1. First, a total of 7 copies of 37 bp to 40 bp tandem repeats are observed within the *repA* iterons of pIMP-HZ1, while only 3 copies are found in pP378-IMP and p1220-IMP (Fig. 3). Second, pP378-IMP and IMP-HZ1 contains an intact antirestriction system *ecoRII*-*ecoRIImet* (located around 8.5 kb to 11.5 kb nucleotide position of pP378-IMP), while only a truncated *ecoRIImet* gene is found in p1220-IMP and this truncation likely results from the insertion of IS*Kpn19* upstream (Fig. 3). Third, the inversion of the conjugal transfer region from orf207 to the 3′-end remnant of *fipA* undergone occurs within pP378-IMP and p1220-IMP related to IMP-HZ1 (Fig. 3).

**Figure 4:**
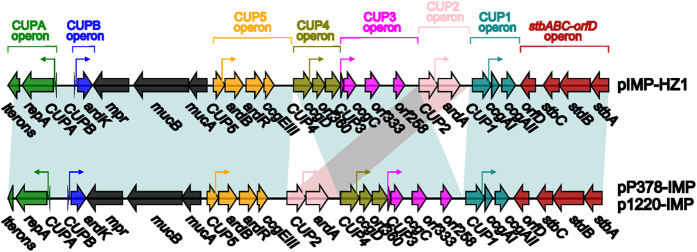
CUP-control regulons Genes are denoted by arrows and colored based on gene function classification. The broken lines with terminal arrows indicate the core promoter regions of indicated operons. Shading regions denote shared DNA regions of homology (>95% nucleotide identity).

The fourth major genetic difference (Fig. 4) is found within the CUP-controlled regulon^10^. A total of four putative operons, namely the CUPA operon, the CUPB operon, the CUP5 operon, the CUP4 operon, the CUP3 operon, the CUP2 operon and the CUP1 operon, are arranged within this regulon; each of these operons contains a putative ArdK-binding site and a promoter, which are responsible for ArdK-dependent expression of corresponding genes^10^. Compared with pIMP-HZ1, the translocation of the CUP2 operon occurs within pP378-IMP and p1220-IMP, which most likely results from the homologous recombination mediated by CUP1, CUP2 and CUP4 (Fig. 4).

### Accessory modules of pP378-IMP and p1220-IMP

**Figure 5:**
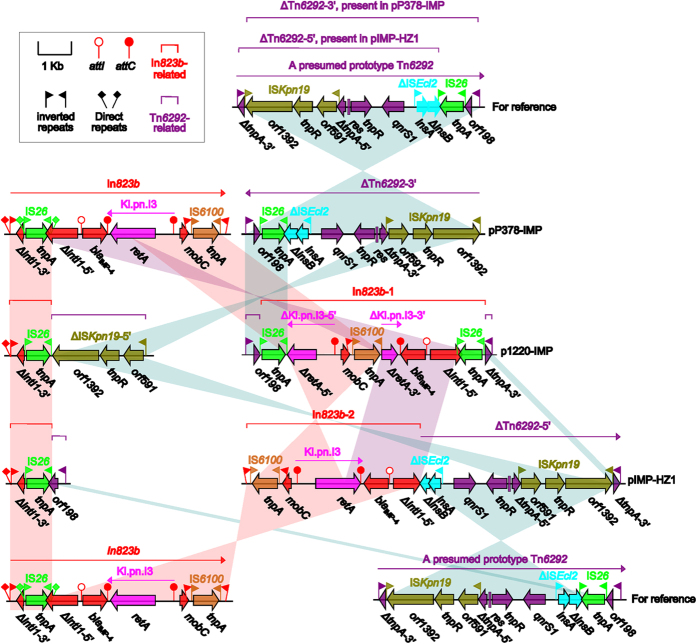
Plasmid accessory resistance regions. Genes are denoted by arrows and colored based on gene function classification. Shading regions denote regions of homology (>95% nucleotide identity).

pP378-IMP carries three separate accessory modules, a 6492 bp class 1 integron designated In*823b*, a 290 bp IS*1* remnant, and a 7075 bp truncated version (designated Δ*Tn6292*-3′) of a presumed Tn*3*-family unit transposon Tn*6292* (Fig. 5).

The 290 bp IS*1* remnant, which contains only Δ*insB* (transposase) and inverted repeat right (IRR) and is inserted between *ecoRIImet* and *orf207*, is shared by pP378-IMP, p1220-IMP and IMP-HZ1 (Fig. 1).

Multiple copies of IS26 are present in the In*823b*- and Tn*6292*-related regions of pP378-IMP, p1220-IMP and IMP-HZ1, and the common component IS*26* would act as an adaptor^11^,^12^ to mediate massive fragmentation and rearrangements of In*823b*- and Tn*6292*-related regions in p1220-IMP and IMP-HZ1 relative to pP378-IMP (Fig. 5), leaving different mosaic assemblies from the remnants of In*823b* and Tn*6292* in p1220-IMP and IMP-HZ1. Nevertheless, all these accessory genetic contents are integrated at two “hotspots” (Fig. 1), namely a region downstream of *resP* (resolvase) and a region within *fipA* (fertility inhibition protein), which has been previously described in IncN1 plasmids^2^,^4^.

Compare with the fragmentary In*823b*-related regions in pIMP-HZ1 and p1220-IMP, the In*823b* integron from pP378-IMP looks like a primitive form flanked by a complete set of inverted repeats (IRs, 25 bp in length) and direct repeats (DRs, 5 bp in length: target site duplication signals of transposition) (Fig. 5)^13^. The 5′-conserved segment [5′-CS: IRi (inverted repeat initial)-*intl1* (integrase)-*attI*] of In*823b* is disrupted by the insertion of IS*26* into *intl1*. In*823b* contains a single resistance gene cassette *bla*_IMP-4_*-attC*_*bla*IMP-4_, and a group IIc intron Kl.pn.I3 disrupts an unusual *attC* site that appears to be a chimera between *attC*_*bla*IMP-4_ and *attC*_*dfrA14GC*_. Downstream of *attC*_*dfrA14GC*_ is a structure *mobC* (Mobilization protein)-IRi-IS*6100*-IRt (inverted repeat terminal), but the typical 3′-conserved segment [3′-CS: *qacED1* (quaternary ammonium compound resistance)-*sulI* (sulfonamide resistance)-IRt] in not found. The expression of *bla*_IMP-4_ is driven by a single promoter PcW_TGN-10_. which is a derivate of the weak promoter PcW and much stronger than PcW due to the C to G mutation 2 bp upstream of the −10 element^14^.

Two Tn*6292*-related fragments (namely ΔTn*6292*-3′ and ΔTn*6292*-5′) are present in pP378-IMP and pIMP-HZ1, respectively, with a large overlapping region between these two plasmids, which promotes us to propose a prototype Tn*3*-family unit transposon Tn*6292*, 7314 bp in length, with typical 38 bp IRs (IRL: inverted repeat left; IRR: inverted repeat right) at both ends (Fig. 5). The Tn*6292* core transposition module *tnpA* (transposase)-*res* (resolution site)-*tnpR* (resolvase) is disrupted by the insertion of IS*Kpn19* into *tnpA*, leaving it truncated and broken into two separate parts Δ*tnpA*-3′ and Δ*tnpA*-5′; downstream of *tnpR* is a quinolone resistance region *qnrS1*-ΔIS*Ecl2*-IS*26*-*orf198*. The *qnrS1* gene and its upstream insertion sequence ΔIS*Ecl2* constitute a core *qnrS1* genetic platform that is widely found in resistance plasmids from *Enterobacteriaceae* species, and it is thought that ΔIS*Ecl2* could have played a role in the original acquisition of *qnrS1*^15^,^16^.The ΔTn*6292*-3′ element of pP378-IMP is a 7075 bp 3′-region of Tn*6292* lacking IRL-Δ*tnpA*-3′, while ΔTn*6292*-5′ from pIMP-HZ1 is a 6048 bp 5′-region of Tn*6292* in the absence of IS*26*-*orf198*-IRR (Fig. 5).

Compared to In*823b* and Tn6292-3′ from pP378-IMP, massive fragmentation of these two accessory regions, followed by further inversion and translocation of the resulting In*823b*- and Tn*6292*-derived fragments, occurs in p1220-IMP and IMP-HZ1, leaving the assembly of different combinations of accessory regions with a very complex mosaic nature in these two plasmids (Fig. 5). pP378-IMP contains a total of two resistance genes *bla*_IMP-4_ and *qnrS1*, which are captured by In*823b* and ΔTn*6292*-3′, respectively. *bla*_IMP-4_ is also present in the In*823b*-derived elements In*823b*-1 and In*823b*-2 (which can be discriminated as the partial regions of In*823b*) from p1220-IMP and IMP-HZ1, respectively. *qnrS1* is also present in ΔTn*6292*-5′ from pIMP-HZ1, but it not found in p1220-IMP.

### Prevalence of pP378-IMP/p1220-IMP-related plasmids

A total of 12 backbone genes *repA, mrr, kikA, traL, traB, traF, traJ, stdB, ccgAII, ardA, mucB*, and *ardK* as well as the accessory quinolone-resistance gene *qnrS1* were arbitrarily selected for PCR detection, followed by amplicon sequencing (data not shown). It was found that all these 12 backbone genes were present in all the *bla*_IMP-4_-carrying 19 *K. pneumoniae* strains and 7 *P. aeruginosa* strains (Table S1). The above results indicated that pP378-IMP/p1220-IMP-like plasmids were harbored in all these *bla*_IMP-4_-carrying *K. pneumoniae* and *P. aeruginosa* strains. The *qnrS1* gene was detected in 3 *bla*_IMP-4_-carrying *K. pneumoniae* strains and in 4 *bla*_IMP-4_-carrying *P. aeruginosa* strains (Table S1), denoting the probable coexistence of the In*823*-derived *bla*_IMP-4_ regions and the Tn*6296*-derived *qnrS1* regions in these strains.

## Methods

### Bacterial strains and identification

Bacterial species was identified by 16S rRNA gene sequencing^17^ and by PCR detection of *K. pneumoniae*-specific gene *khe*^18^, *P. aeruginosa*-specific *oafA*^19^ and *A. baumannii*-specific *bla*_OXA-51_^20^. The major plasmid-borne carbapenemase and extended-spectrum β-lactamase genes were screened for by PCR^21^, followed by amplicon sequencing on ABI 3730 Sequencer (LifeTechnologies, CA, USA). The MLST schemes for *K. pneumoniae* and *P. aeruginosa* were derived from the PubMLST database (http://pubmlst.org/).

### Plasmid conjugal transfer

Plasmid conjugal transfer experiments were carried out with the rifampin-resistant *Escherichia coli* EC600 (LacZ^−^, Nal^R^, Rif ^R^) being used as recipient and strain P378 or 1220 as donor. 3 ml of overnight culture of each of donor and recipient bacteria were mixed together, harvested and resuspended in 80 μl of Brain Heart Infusion (BHI) broth (BD Biosciences). The mixture was spotted on a 1 cm^2^ filter membrane that was placed on BHI agar (BD Biosciences) plate, and then incubated for mating at 37 °C for 12 to 18 h. Bacteria were washed from filter membrane and spotted on Muller-Hinton (MH) agar (BD Biosciences) plate containing 1000 μg/ml rifampin and 2 μg/ml imipenem for selection of *bla*_IMP_-positive *E. coli* transconjugants.

### Detection of carbapenemase activity

Activity of class A/B/D carbapenemases in bacterial cell extracts was determined via a modified CarbaNP test^21^. Overnight bacterial cell culture in MH broth was diluted 1:100 into 3 ml of fresh MH broth, and bacteria were allowed to grow at 37 °C with shaking at 200 rpm to reach an OD_600_ of 1.0 to 1.4. If required, ampicillin was used at 200 μg/ml. Bacterial cells were harvested from 2 ml of the above culture, and washed twice with 20 mM Tris-HCl (pH 7.8). Cell pellets were resuspended in 500 μl of 20 mM Tris-HCl (pH 7.8), and lysed by soniation, followed by centrifugation at 10000 × g at 4 °C for 5 min. 50 μl of the supernatant (the enzymatic bacterial suspension) were mixed with 50 μl of substrate I to V, respectively, followed by incubation at 37 °C for a maximum of 2 h. Substrate I: 0.054% phenol red plus 0.1 mM ZnSO_4_ (pH7.8). Substrate II: 0.054% phenol red plus 0.1 mM ZnSO_4_ (pH7.8), and 0.6 mg/μl imipenem. Substrate III: 0.054% phenol red plus 0.1 mM ZnSO_4_ (pH7.8), 0.6 mg/μl mg imipenem, and 0.8 mg/μl tazobactam. Substrate IV: 0.054% phenol red plus 0.1 mM ZnSO_4_ (pH7.8), 0.6 mg/μl mg imipenem, and 3 mM EDTA (pH7.8). Substrate V: 0.054% phenol red plus 0.1 mM ZnSO_4_ (pH7.8), 0.6 mg/μl mg imipenem, 0.8 mg/μl tazobactam, and 3 mM EDTA (pH7.8).

### Bacterial antimicrobial susceptibility test

Bacterial antimicrobial susceptibility was tested by VITEK 2 (BioMérieux Vitek, Hazelwood, MO, USA) and interpreted as per Clinical and Laboratory Standards Institute (CLSI) guidelines^22^.

### Plasmid sequencing and sequence assembly

Plasmid DNA was isolated from *E. coli* transconjugant using Qiagen large construct kit (Qiagen, Hilden, Germany), and sequenced by whole-genome shotgun strategy in combination with Illumina HiSeq 2500 (Illumina, San Diego, CA, USA) sequencing technology. Reads from each sample were trimmed to remove poor quality sequences, and then the contigs were assembled with Velvet. The gaps were filled through combinatorial PCR and Sanger sequencing on ABI 3730 Sequencer.

### Sequence annotation and genome comparison

The open reading frames and pseudogenes were predicted with GeneMarkS™ (http://topaz.gatech.edu/GeneMark), RAST (http://rast.nmpdr.org/), and Prodigal (http://compbio.ornl.gov/prodigal), and further annotated by BLASTP and BLASTN against UniProtKB/Swiss-Prot (http://web.expasy.org/docs/swiss-prot_guideline.html) and NCBI NR databases. Annotation of resistance genes, mobile elements and other gene futures was based on the relevant databases including CARD (http://arpcard.mcmaster.ca), BacMet (http://bacmet.biomedicine.gu.se/), β-lactamases Database (http://www.ncbi.nlm.nih.gov/pathogens/submit_beta_lactamase), ISfinder (https://www-is.biotoul.fr/), ISCR Elements Databases (http://medicine.cf.ac.uk/infect-immun/research/infection/antibacterial-agents/iscr-elements), INTEGRALL (http://integrall.bio.ua.pt/?), Tn Number Registry (http://www.ucl.ac.uk/eastman/research/departments/microbial-diseases/tn), and Group II Introns Databases (http://webapps2.ucalgary.ca/~groupii/blast.html). Sequence comparison was performed with BLASTN and CLUSTALW, and gene organization diagrams were drawn with Inkscape (https://inkscape.org). The complete sequence of pP378-IMP and p1220-IMP were submitted to GenBank under accession numbers KX711879 and KX711880, respectively.

## Additional Information

**How to cite this article**: Feng, W. *et al*. Dissemination of IMP-4-encoding pIMP-HZ1-related plasmids among *Klebsiella pneumoniae* and *Pseudomonas aeruginosa* in a Chinese teaching hospital. *Sci. Rep.* **6**, 33419; doi: 10.1038/srep33419 (2016).

## Supplementary Material

Supplementary Information

## Figures and Tables

**Table 1:** Antimicrobial drug susceptibility profiles.

Category	Antibiotics	MIC (mg/L)/antimicrobial susceptibility				
		1220	1220-IMP-EC600	P378	P378-IMP-EC600	EC600
Penicillins	Ampicillin	≥32R	≥32R	≥32R	≥32R	8S
	Ampicillin/sulbactam	≥32R	≥32R	≥32R	≥32R	4S
Cephalosporins	Cefazolin	≥64R	≥64R	≥64R	≥64R	≤4S
	Cefuroxime	≥64R	≥64R	≥64R	≥64R	16I
	Cefotetan	≥64R	≥64R	≥64R	≥64R	≤4S
	Ceftriaxone	≥64R	≥64R	≥64R	≥64R	≤1S
	Ceftazidime	≥64R	≥64R	≥64R	≥64R	≤1S
	Cefepime	32R	32R	≥64R	32R	≤1S
Carbapenems	Imipenem	8R	8R	8R	8R	≤1S
	Meropenem	8R	8R	8R	8R	≤0.25S
Fluoroquinolones	Ciprofloxacin	≤0.25S	≤0.25S	≥4R	≥4R	≤0.25S
	Levofloxacin	0.5S	0.5S	≥8R	≥8R	0.5S
Aminoglycosides	Amikacin	≤2S	≤2S	16S	≤2S	≤2S
	Gentamicin	≤1S	≤1S	≤1S	≤1S	≤1/SS
	Tobramycin	≤1S	≤1S	≥16R	≤1S	≤1S
Sulfanilamides	Trimethoprim/sulfamethoxazole	≤20S	≤20S	≥320R	≤20S	≤20S

S = sensitive; R = resistant; I = intermediate.
